# Characterizing Woody Breast Myopathy in a Meat Broiler Line by Heat Production, Microbiota, and Plasma Metabolites

**DOI:** 10.3389/fvets.2019.00497

**Published:** 2020-02-04

**Authors:** Pramir Maharjan, Katie Hilton, Jordan Weil, Nawin Suesuttajit, Antonio Beitia, Casey M. Owens, Craig Coon

**Affiliations:** Department of Poultry Science, Center of Excellence for Poultry Science, University of Arkansas, Fayetteville, AR, United States

**Keywords:** woody breast myopathy, heat production, microbiota, plasma metabolites, meat broiler

## Abstract

Selection for quantitative traits in meat broilers such as breast yield and growth rate exert physiological pressure leading to ante mortem histological and biochemical alterations in muscle tissues. The poultry industry has recently witnessed a myopathy condition affecting *Pectoralis major* (breast muscle) of broilers, called woody breast (WB), an etiology still unclear to scientific community. A study was conducted to characterize the WB myopathy in a meat broiler line at its finishing phase (d 41) in terms of heat production (HP), microbiota and plasma metabolites. Two treatment groups were studied—WB affected (myopathy) and normal (non-myopathy) broiler; *n* = 20 in each group. Indirect calorimetry was utilized for HP measurement. Furthermore, body composition (BC) analysis was also performed using dual-energy x-ray absorptiometry (DEXA). Microbiota in ileal digesta was studied with PCR amplified 16s rRNA gene. LC-MS targeted metabolomics was performed to understand differential expression of plasma metabolites. Results showed that there was difference in fasting HP (*P* < 0.05) between these two treatment groups, with non-myopathy broiler producing more heat which was indicative of higher body protein content validated by higher protein: fat ratio by BC results. Less protein content in myopathy bird could be due to probable higher mixed muscle degradation occurring in lean tissue as marked by elevated 3-methylhistidine expression in plasma. Microbiota results showed unclassified *Lactobacillus* as predominant genus with higher abundance occurring in myopathy group; whereas at species level, *L. acidipiscis* was predominant bacteria for non-myopathy broiler. Differentially significant metabolites (*P* < 0.05) identified from plasma metabolome between these two treatment groups were homocysteine, cyclic GMP, trimethylamine N-oxide (TMAO), tyramine, carnitine, and acetylcarnitine, which were all associated to cardiovascular system. The findings suggest that more research in meat broilers could be opted toward delivering reduced vascularity issues to alleviate this myopathy condition.

## Introduction

Progressive improvements in genetic selection and nutritional strategies in meat broilers to grow them faster have exerted these broiler strains subject to various physiological stresses (1, 2). This has also resulted in emergence of several muscle myopathies including one called woody breast (WB) myopathy. This myopathy condition affects the *Pectoralis major* (poultry breast) muscle (3, 4). A recent study reported WB myopathy incidence of ~9% for 10,483 filets evaluated in high breast meat yielding strain from a flock which produced larger broilers (2.72–4.53 kg) (5). The WB affected filet is phenotypically stiff and visually unappealing along with reduced protein quality. Histologically, it has been reported to exhibit moderate to severe polyphasic myodegeneration with variable degrees of interstitial connective tissue accretion or fibrosis (6).

The exact biological mechanism for causation of this myopathy is still unclear to scientific community, while many studies have been conducted particularly in past 5-year span to understand its associated etio-pathophysiological processes (3, 7, 8). This study is another attempt to understand myopathy bird from non-myopathy ones with the goal of characterizing physiological perturbations undergoing in this diseased condition. Here, we were interested in characterizing heat production (**HP**) and body composition (**BC**) differences in these two groups to understand if there is disparity in dietary nutrient utilization and its partitioning in myopathy state. We utilized metabolic chambers to measure HP, and Dual Energy X-ray Absorptiometry (**DEXA**) to predict BC. This is the first study undertaken to characterize myopathy and non-myopathy broilers for HP changes to our knowledge. Simultaneously, we also evaluated microbiota differences in ileal digesta, and measured differential expression of plasma metabolites to uncover associated biomarkers in comprehending possible etio-pathology involved in this myopathy condition.

## Methods

### Bird Type, Husbandry, and Sampling

The experiment was conducted in a fast-growing commercial meat type broiler strain, *Gallus gallus domesticus*, in its finishing grow-out phase, grown feeding in floor pens with recommended nutrient guidelines as per the strain requirement (Table 1). Broilers were randomly selected and scored for WB myopathy from the scale of 0–3, 0 being the non-myopathy broiler and 3 being the severely affected myopathy broiler (9) at d 41. Therefore, two treatment groups- myopathy and non-myopathy were created. For each group of non-myopathy (WB score < 1) and myopathy (*n* ≥ 2), *n* = 20 birds were selected with 40 birds utilized in the study. Twelve broilers, each treatment group, were then utilized for HP study. Remaining eight broilers for each category were then utilized for BC study. Bird used for the BC study were also sampled for histology (breast tissue), ileal digesta and blood samples. Tissue sample was fixed in 10 % formalin solution until subjected for histological slide preparation (Masson Trichome staining) and visualized using light microscopy (results only mentioned in discussion section). Ileal digesta (*n* = 8 each group) collected was immediately frozen with liquid N2. Plasma (*n* = 8 each group) was separated from blood samples. Digesta and plasma samples were then stored at −79°C until analysis. Ileal digesta were subjected for microbiota study, whereas blood samples were utilized for plasma metabolite analysis. The procedures for microbiota and metabolite study are explained in detail in the methods below.

**Table 1:** Experimental finisher diet composition.

**Ingredient**	**%**
Corn	66.66
SBM	27.89
Dicalcium phosphate	1.49
Limestone	0.82
Corn oil	1.96
Salt	0.35
DL-Methionine	0.23
L-lysine	0.15
L-Threonine 98%	0.01
Trace mineral	0.1
Vitamins premix	0.1
Ark phytase	0.05
Kemin Mold curb-50%	0.05
Selenium premix-0.06%	0.02
Monsanto sanoquin 6 etho	0.02
**Calculated values**	
ME (Kcal/kg)	3,100
CP	18.77
Dig Lysine	1.00
Dig Methionine	0.49
Dig C+M	0.76
Dig Threonine	0.65

*Supplies per kilogram of diet: antioxidant, 200 mg; retinyl acetate, 21 mg; cholecalciferol, 110 μg; D-α-tocopherol acetate, 132 mg; menadione, 6 mg; riboflavin, 15.6 mg; D-calcium pantothenate, 23.8 mg; niacin, 92.6 mg; folic acid, 7.1 mg; cyanocobalamin, 0.032 mg; pyridoxine, 22 mg; biotin, 0.66 mg; thiamine, 3.7 mg; choline chlorine, 1,200 mg; Mn, 100 mg; Mg, 27 mg; Zn, 100 mg; Fe, 50 mg; Cu, 10 mg; I, 1 mg; Se, 200 μg. Axtra Phytase. Included at a rate of 50 g/MT to the basal diet to supply a guaranteed minimum of 500 FTY/kg of feed*.

### Heat Production and Body Composition

#### Heat Production

Heat production was measured following the principle of indirect calorimetry system utilizing respiratory chambers. Each chamber (*n* = 2 birds) acted as a replicate of a treatment group. There were six replicates for each treatment group, with 12 chambers being utilized in the study. The same respiratory chambers units described in Caldas et al. (10) were utilized in the study to measure oxygen consumption (VO_2_) and carbon dioxide (VCO_2_) production per day at 41–43 d. The birds after moving to chambers were acclimatized for 24 h. The calibration of the gas measurement chamber units was performed at the beginning of experiment. Feed consumed per bird were accounted during the experimental period. The gas evaluation in each chamber was measured every 12 min, so every chamber unit provided 5 readings for 1 h, totaling 120 readings per day. Fed HP and fasting HP were measured each for 24 h utilizing the equations described below.

The VO_2_ and VCO_2_ obtained in liter (L)/d was the utilized to calculate HP using the following equation (11): HP, Kcal/d = 3.866 VO_2_ L/d + 1.233 VCO_2_ L/d.

Heat production was then adjusted to per unit metabolic BW per day. Heat increment (HI) was also accounted as HI = Fed HP- fasted HP.

#### Body Composition

Broilers (*n* = 8) selected were scanned for BC study using DEXA equipped with Lunar Prodigy small animal software. Scanned values were used in previously determined equations (12) to calculate BC in terms of protein mass, and fat mass in treatment broilers; and protein to fat ratio were recorded.

#### Data Analysis

The data obtained for HP and BC were analyzed by one-way ANOVA using JMPro 14 (SAS Institute, Inc., Cary, NC). Mean values were obtained for variables measured (HP, and protein to fat mass ratio). Significant means for the measured variables were separated using student's *t*-test or HSD test where appropriate. Means were considered significant for *P* ≤ 0.05.

### Microbiota Analysis

#### DNA Extraction, PCR, Sequencing, and Sequence Processing

Bacterial DNA were extracted from ileal digesta using MoBio PowerMag Soil DNA Isolating Bead Plate. Following DNA extraction, bacterial 16S rRNA genes were PCR-amplified with dual-barcoded primers targeting the V4 region (515F 5′-GTGCCAGCMGCCGCGGTAA-3′, and 806R 5′-GGACTACHVGGGTWTCTAAT-3′ (13). Using the 300-bp paired-end kit (v.3), amplicons were then sequenced with an Illumina MiSeq. Sequences obtained were denoised, and taxonomically classified using Greengenes (v. 13_8) as the reference database. Classified sequences were clustered into 97%-similarity operational taxonomic units (OTUs) with the mothur software package (v. 1.39.5) (14). The potential for contamination was addressed by co-sequencing DNA amplified from specimens and from 2 each of template-free controls and extraction kit reagents processed the same way as the specimens. Operational taxonomic units were considered putative contaminants (and were removed) if their mean abundance in controls reached or exceeded 25 % of their mean abundance in specimens.

#### Alpha and Beta Diversity Measurements and Data Analysis

Shannon index on raw OTU abundance tables were utilized to estimate alpha diversity. The significance of diversity differences was tested with ANOVA. To estimate beta diversity across samples, OTUs occurring with a count of <3 in at least 10% of the samples were excluded and then computed Bray-Curtis indices. Beta diversity was visualized emphasizing differences across samples, using Principal Coordinate Analysis (PCoA) ordination. Variation in community structure was assessed with permutational multivariate analyses of variance (PERMANOVA) with treatment group as the main fixed factor and using 9,999 permutations for significance testing. The analyses were conducted in the R environment.

### LC-MS Targeted Metabolomics

Targeted metabolomics of polar, primary metabolites was performed on a TQ-XS triple quadrupole mass spectrometer (MS) coupled to an I-class UPLC system (Waters) for differential expression. Plasma was thawed on ice then 80 μL was aliquoted to a 1.5 mL Eppendorf tube on ice. Next, 240 μL of ice-cold methanol was added and samples were vortexed to mix. Samples were incubated for 30 min at −80° C, and then centrifuged at 16,000 × g at 4°C for 15 min. The supernatant was analyzed by LC-MS. Separations were carried out on a ZIC-pHILIC column (2.1 × 150 mm, 5 μM) (EMD Millipore). The mobile phases were (A) water with 15 mM ammonium bicarbonate adjusted to pH 9.6 with ammonium hydroxide and (B) acetonitrile. The flow rate was 200 μL/min and the column were held at 50°C. The injection volume was 1 μL. The gradient was as follows: 0 min, 90% B; 1.5 min, 90% B; 16 min, 20% B; 18 min, 20% B; 20 min, 90% B; 28 min, 90% B. The MS was operated in selected reaction monitoring (SRM) mode. Source and desolvation temperatures were 150 and 600°C, respectively. Desolvation gas was set to 1,100 L/h and cone gas to 150 L/h. Collision gas was set to 0.15 mL/min. All gases were nitrogen except the collision gas, which was argon. Capillary voltage was 1 kV in positive ion mode and 2 kV in negative ion mode.

#### Data Processing and Bioinformatics Analyses

Data obtained were normalized to total ion abundance. Coefficient of variance >30% in the quality control injections were removed. Mass spectral metabolite databases were searched against including Metlin, Mass Bank of North America, and an in-house database. Data was processed using Skyline software (15).

## Results

### Heat Production and Body Composition

#### Heat Production

**Figure 1:**
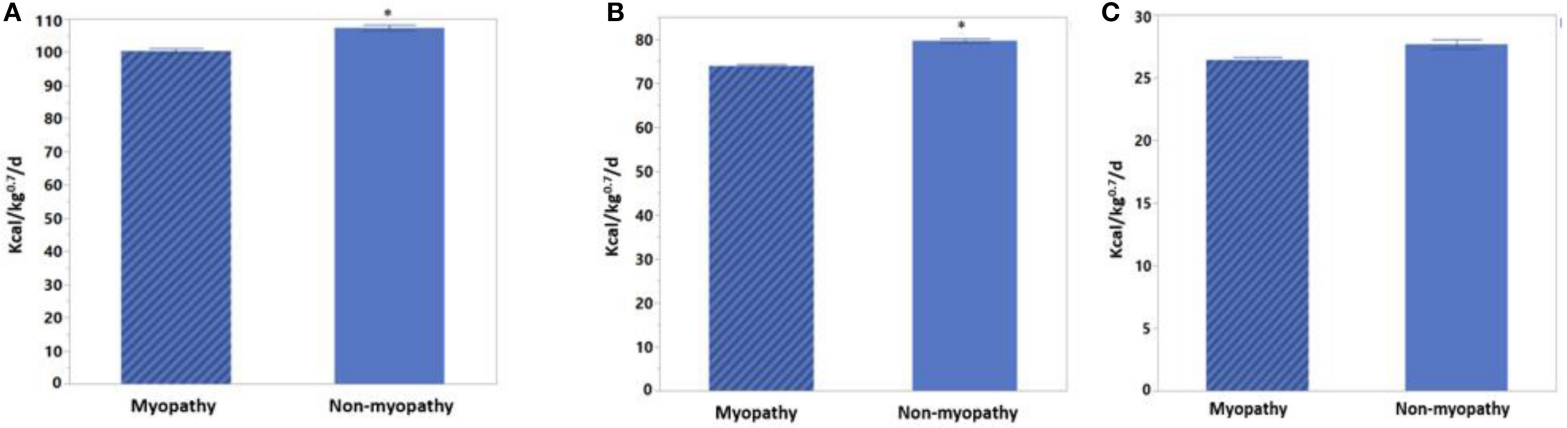
Heat production for myopathy and non-myopathy broiler at finishing phase (d 41). **(A)** Fed HP **(B)** Fasted HP **(C)** Heat increment. Asterisk on the top of the bar represent significanlty different mean values.

Heat production changes between myopathy and non-myopathy birds are given in Figures 1A–C. Fed HP was 100.32 Kcal/kg^0.75^/d for myopathy broiler whereas it was 107.29 Kcal/kg^0.75^/d for non-myopathy (*P* > 0.05). Fasting HP was 73.91 Kca//kg^0.75^/d for myopathy broiler whereas it was higher for non-myopathy 79.63 Kcal/kg^0.75^/d (*P* < 0.05). Heat increment was 27.66 Kcal/kg^0.75^/d for non-myopathy and 26.41 Kcal/kg^0.75^/d for myopathy broiler. RER recorded for fed and fasted states were 0.8795 and 0.8731, and 0.7269 and 0.722070 for myopathy and non-myopathy, respectively.

**Figure 2:**
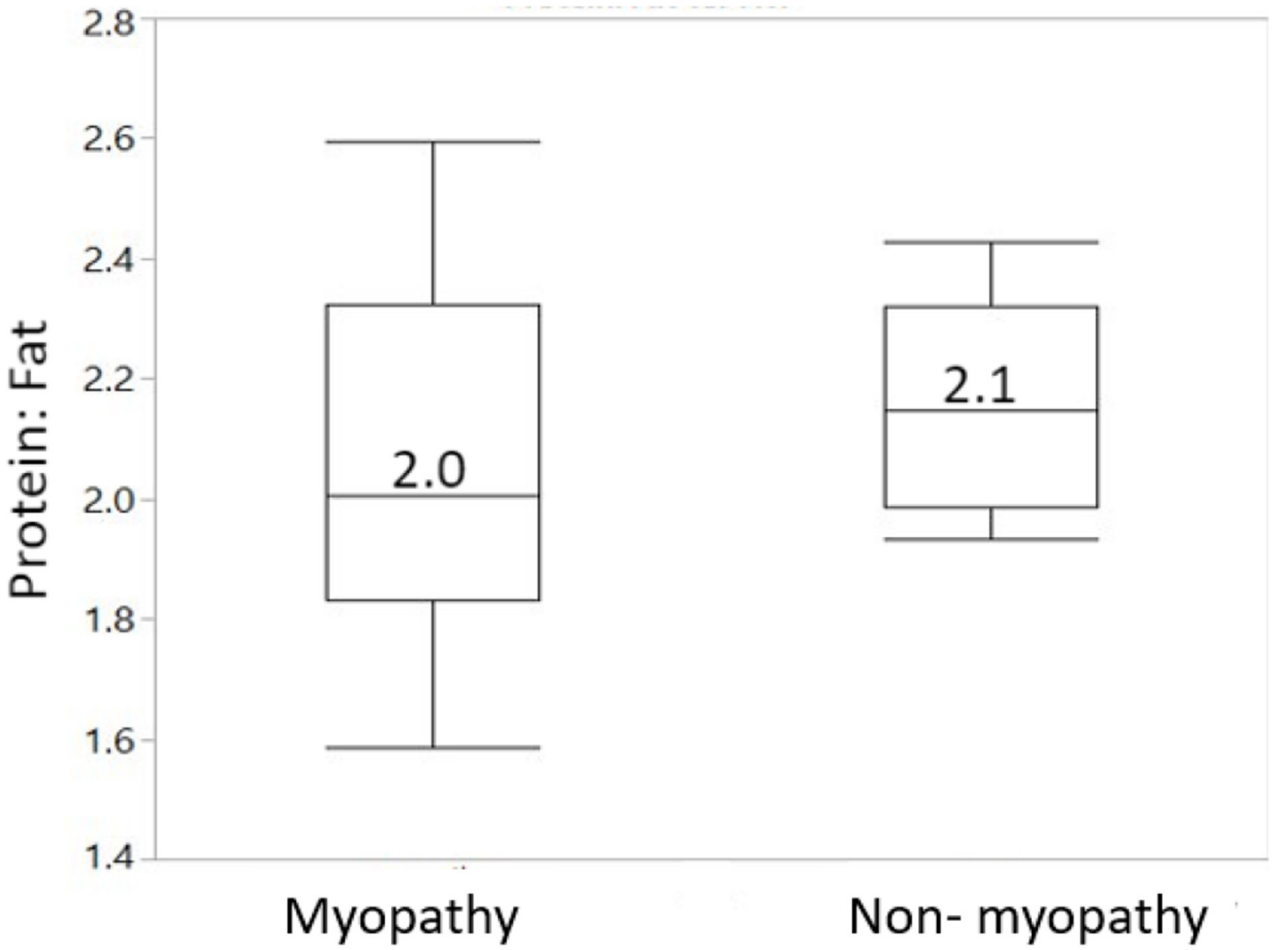
Body composition in terms of protein to fat ratio for myopathy and non-myopathy broiler. *N* = 8 for each treatment group.

Figure 2 gives body composition data for myopathy and non-myopathy birds in terms of protein: fat ratio. The median value of protein to fat ratio for myopathy was 2.0 whereas it was 2.1 for non-myopathy.

### Microbiota

**Figure 3:**
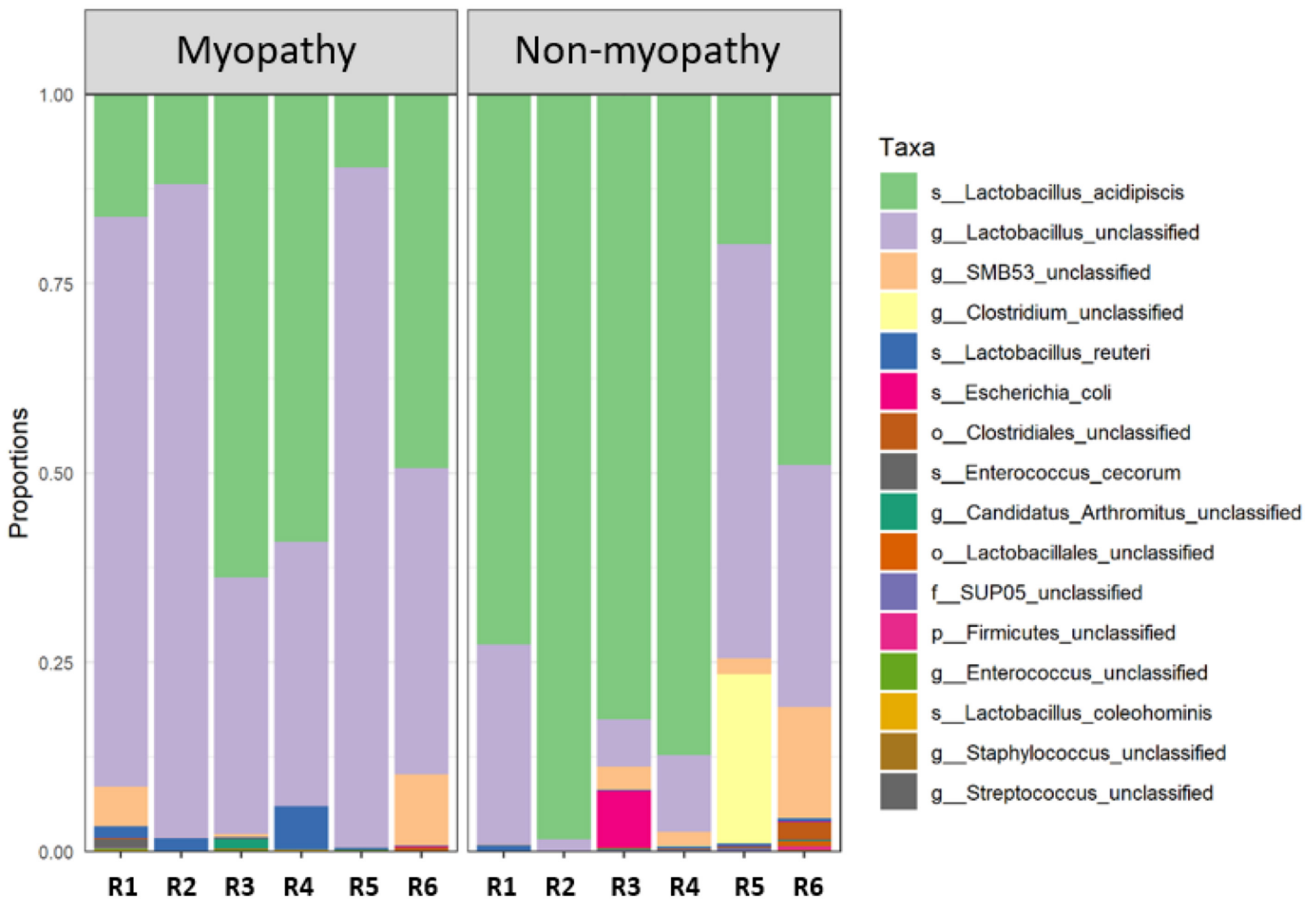
Taxonomic bar plot. Each bar (R1–R6 replicate samples) for myopathy (M) and non-myopathy (NM) represents distribution of the aggregated taxa for each taxonomic rank in proportions. s, species; g, genus; o, order; f, family; and p, phylum. Non-myopathy group has relatively higher abundance of *Lactobacillus acidipiscis* (NM = 68.16%; and M = 34.90% of total abundance); whereas myopathy group has higher proportion of unclassifed generic *Lactobacillus* (M = 60.09%; and NM = 21.84% of total abundance).

**Figure 4:**
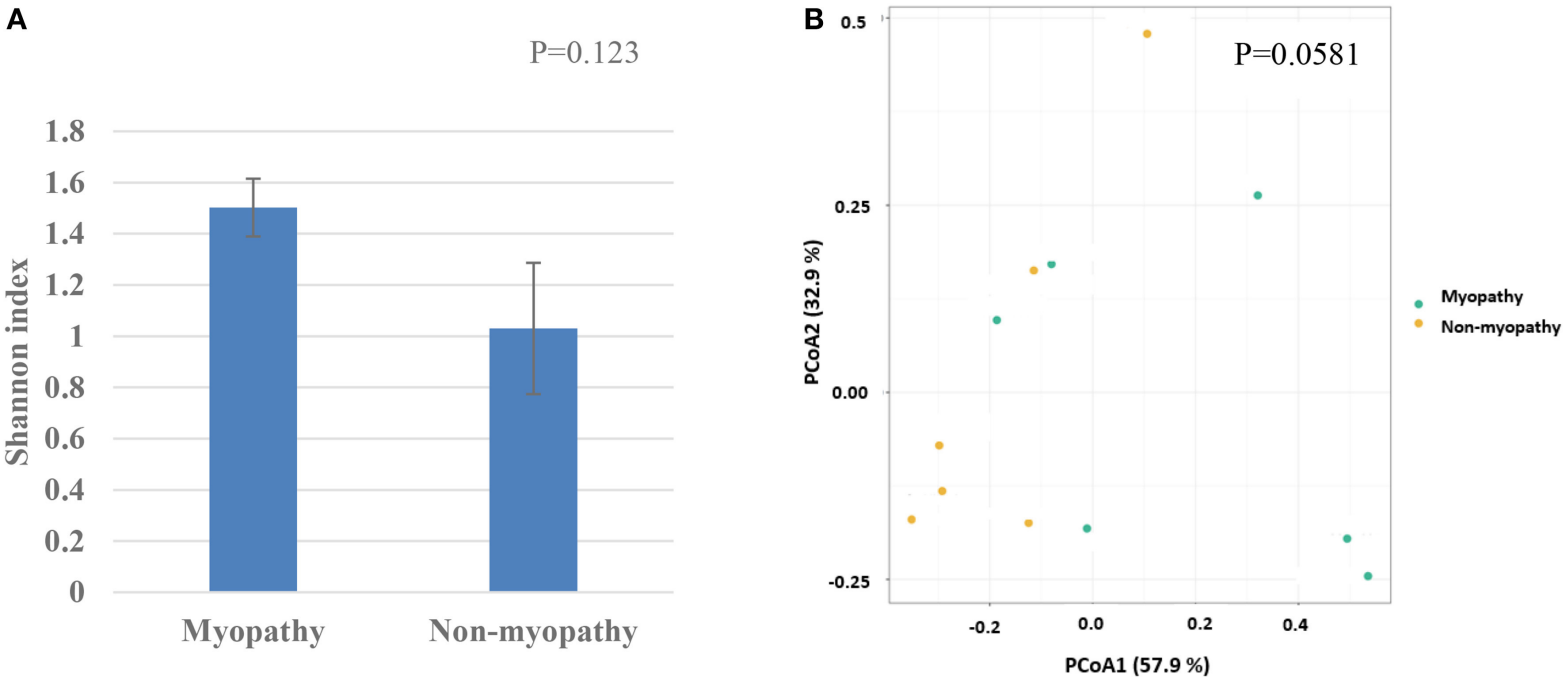
**(A)** Alpha diversity as given by Shannon diversity index for myopathy and non-myopathy broiler. **(B)** Beta-divesity or PCoA ordination as performed by Bray-Curtis dissimilarities.

At phylum level, the predominant taxa identified were firmicutes in both myopathy (99.93%) and non-myopathy (98.56%) groups, followed by proteobacteria, 0.043% and 1.414%, and actinobacteria 0.021 and 0.0157%, respectively. Further, *Lactobacillus* was single most predominant species occupying more than 90% of bacterial population for both the treatment groups. At genus level, unclassified *Lactobacillus* was predominant (60.09%) with myopathy group whereas it was 21.84% in non-myopathy. At the species level, *L. acidipiscis* was predominant bacteria for non-myopathy birds (68.16%) (Figure 3). Alpha diversity was numerically higher for myopathy birds than non-myopathy as indicated by Shannon Diversity Index (Figure 4A). Beta diversity was near to significance (*P* = 0.058) with myopathy birds exhibiting greater sample-to-sample dissimilarity (Figure 4B).

### Differential Expression of Plasma Metabolites

**Figure 5:**
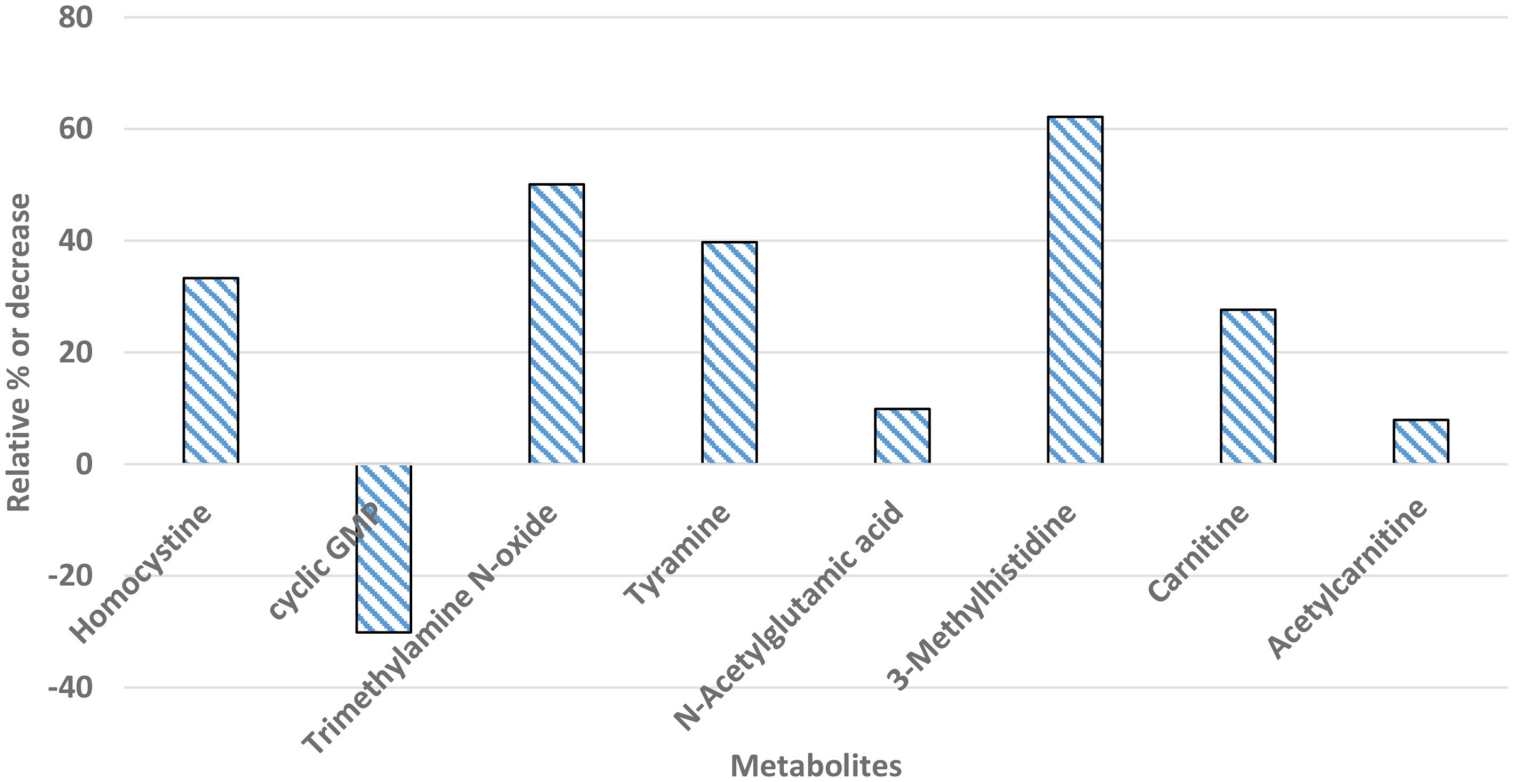
Percent increase or decrease for differentially significant (*P* < 0.05) metabolites in plasma samples of myopathy bird in relation to non-myopathy.

Differentially significant metabolites captured in plasma samples of myopathy and non-myopathy broiler were presented in Figure 5. Differentially significant metabolites (*P* < 0.05) identified between these two treatment groups were homocysteine, cyclic GMP, trimethylamine N-oxide (TMAO), tyramine, carnitine, and acetylcarnitine, which were all associated to cardiovascular system. Homocysteine, TMAO, tyramine, carnitine, and aceltycarnitine were elevated in myopathy-affected broilers, whereas cyclic GMP was higher in non-myopathy group. Three-methyl histidine, a marker that is related to myofibrillar muscle breakdown, was expressed higher in plasma in myopathy bird than non-myopathy.

## Discussion

Various metabolic disorders are evident in meat broilers, mainly arising from adverse response to vast genetic advancement in growth rate and meat yield. Of many metabolic and physiological perturbations that meat broilers encountered, it was predicted that skeletal muscle related metabolic disorder could remain as a major challenge for poultry industry (16). The industry is witnessing the myopathy condition, called WB myopathy, which is of idiopathic origin. In this study, we characterized HP differences utilizing indirect calorimetry between myopathy and non-myopathy type broilers while simultaneously studying BC. In indirect calorimetry, gas exchange (VO2 and VCO2) is measured as result of oxidation of energy substrates, and HP is then calculated from the substrate oxidation stoichiometry. Higher HP (fed or fasted) measured for non-myopathy bird was higher (*P* < 0.05) could be due to higher the maintenance energy requirement for non-myopathy bird than myopathy bird. This greater maintenance HP could be coming from the higher protein content in body as indicated by higher protein: fat ratio in non-myopathy bird; as maintaining greater basal protein amount would require greater energy (17, 18). Lower body protein content in myopathy bird could be arising from higher skeletal mixed muscle protein degradation rates occurring in these type of birds (19) which was also exhibited by higher 3-methylhistidine expression in plasma in this study. Since there were no difference in HI *(P* > 0.05), the ME or NE values contributed from dietary macronutrients remained similar for both the groups.

The RQ, which is the ratio of between the volumes of carbon dioxide produced to the volume of oxygen used, does depend upon the metabolic rate and physiological status of animal. Higher RER values for myopathy broiler could be the outcome of more protein being oxidized or degraded than non-myopathy (20). RQ of above 0.8 in fed state in both groups showed that dietary calorie values given to these birds were sufficient, whereas ~0.72 for both groups at the unfed state possibly indicate oxidation of ethanol, ketones or lipolysis occurring (21). Walsberg and Wolf (22) also reported the RQ value of 0.71 at fasted state which was attributed to incomplete oxidation of fats or non-pulmonary loss of CO2 in the form of bicarbonates.

Microbial characterization showed that there was variation in the abundance of particular genus or species in ileal digesta. *Lactobacillus* that was identified as the predominant genus in this study is ubiquitously inhabiting in nutrient rich ecological niche with more than 200 species and sub species identified (23), which in another study even reported its potential presence in filet post processing and storage (24). In myopathy broiler, it could be possible that genus *Lactobacillus* could be going through the greater level of genetic transformation resulting in presentation of higher proportion of unclassified *Lactobacillus* species. Whereas*, L. acidipiscis* was clearly an abundant *Lactobacillus* species in ileal digesta in non-myopathy bird. Prior research done in *L. acidipiscis* in broilers showed the probiotic potential and higher feed efficiency improvement (25). The species richness was tended to be higher for myopathy group as exhibited by higher value of alpha diversity index; which further could be associated with greater sample-to-sample species variation in myopathy broiler as explained by beta diversity index.

Significant plasma metabolites detected through differential metabolic screening suggested the potential involvement of vascular system in WB myopathy bird. Elevated homocysteine is associated as marker for cardiovascular disease (26) and can be atherogenic and thrombogenic (27). Trimethylamine N-oxide also has been associated as pro-atherogenic in humans. It is generated from oxidized trimethylamine (TMA) occurring in gut microbiota, while the precursors for TMA in gut are mainly dietary betaine, L-carnitine, and choline. Microbiota participate in overall immune function and nutrient metabolism of host. The difference in bacterial phylum firmicutes (~1.55%) between myopathy and non-myopathy broilers could be one potential reason for the resultant difference in TMAO level in plasma (28). Similarly, another significant plasma metabolite identified was tyramine, which was higher in myopathy broiler. Tyramine is biogenic amine and is a decarboxyted product of tyrosine amino acid. It has both immunological and cardiovascular effects for differences in concentration at pico-nanomolar concentration (29). Tyramine concentration is affected by dietary as well GI bacteria (tyramine-producing bacteria e.g. *Lactobacillus*), and higher concentration of tyramine is associated with hypertension in humans (29). The hypertensive and atherogenic effects (of metabolites) could compromise the blood supply in various body tissues including in muscle tissue leading to atrophy and degradation, which could be pathophysiological condition occurring in myopathy bird. As a probable adaptive response to this vascular issue, cyclic GMP was found to be expressed less; the consequence could lead to relaxation of muscle and improvement in vasodilation and circulation (30). Similarly, other adaptive molecules expressed against the hypertensive and atherogenic effects were carnitine and acetyl carnitine. These compounds are involved in reduction intracellular buildup of toxic metabolites in ischemic condition (31). Plasma metabolic changes observed in this study in myopathy-affected broiler can be associated with findings from genomic and histological data. A recent study performed in broiler where they studied time series differential expression of genes in WB affected *P. major* using RNAseq showed molecular perturbations involving vasculature in early pathogenesis (32). Similarly, another study found the genes related to hypoxia were upregulated in WB affected muscle fibers (33), and microscopical morphometry of vessel density in muscle showed the compromised blood supply in affected tissue (34). Additionally, *P. major* histological or compositional analysis have exhibited increased lipid content in tissue as an important attribute of muscle myopathy (6, 35–38). This increased lipidosis in WB affected muscle could be arising from the event of vascular complexity in meat broilers.

**Figure 6:**
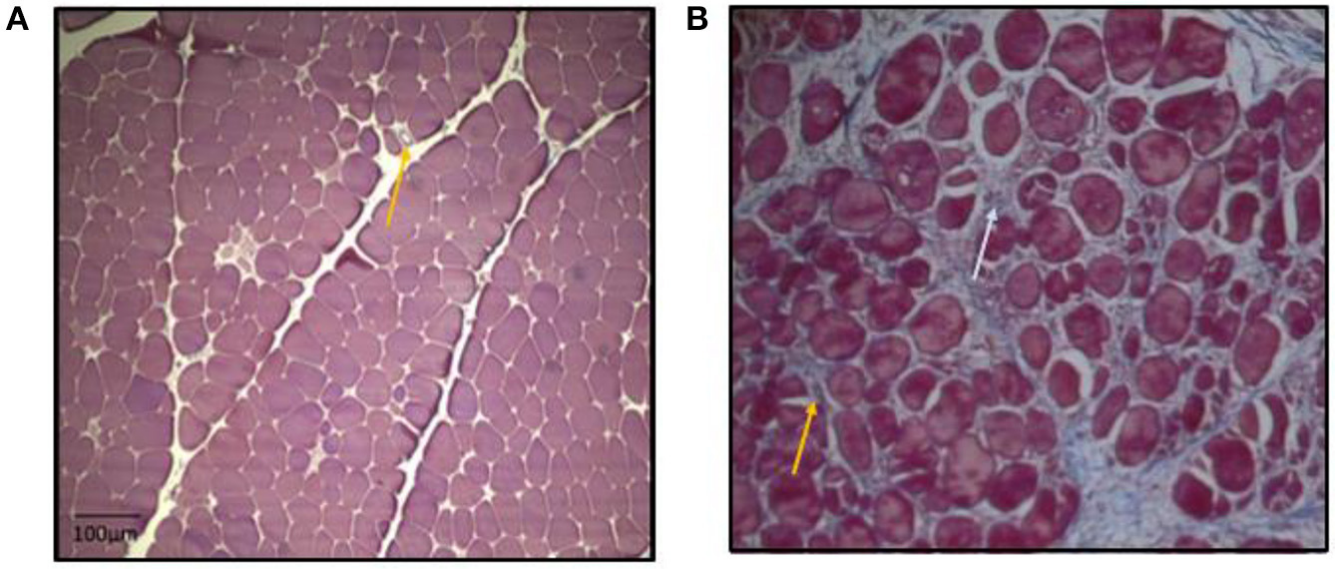
Micrographs: **(A,B)** Cross-section of *P. major* muscle, MT staining. Micrograph **(A)** has less myodegeneration (non-myopathy broiler) than micrograph **(B)** (myopathy broiler). Micrograph **(A)**: day 41 micrograph demonstrating tightly packed polygonal fibers with a minimal extracellular perimysial and endomysial connective tissue spaces. Connective tissue spaces are wider in micrograph **(B)** as compared to micrograph **(A)**, and the spaces tend to be filled with strands of collagenous tissue (bluish coloration as indicated by white arrow). Greater variation in shape and size of fibers were observed in micrograph B. Relatively more fibrosed and constricted veins in micrograph **(B)** than in micrograph **(A)** (as indicated by yellow arrows).

In summary, higher growth rate of muscle fibers in breast muscle in modern meat broiler could possibly aid in narrowing down available space (perimysial and endomyisial connective tissue spaces) between muscle fiber bundles thus also limiting the space available for capillaries. This can potentially lead to inadequate removal of metabolic intermediate products, reduced oxygen supply, reduced fiber functionality and increased degenerative myopathy. While microbiota shifts could be one contributing factor in changes in plasma metabolome, the differential metabolic shifts in plasma of myopathy-affected broiler suggested ongoing vascularity issue delivering chronic localized hypoxic condition and subsequent muscle atrophy in P. major. These atrophied muscle specific protein was replaced by collagenous tissue as depicted by histomicrograph results (Figure 6). These pathophysiological and histochemical phenomena occurring in myopathy broiler have caused net loss of muscle protein quality, and quantity, which was evidenced BC, and HP changes particularly for maintenance or fasting HP. The findings of this study warrant the investigations be directed toward improving meat broiler lines that will deliver improved vascularity to alleviate this myopathy condition.

## Data Availability Statement

Data can be made available upon request to the corresponding author.

## Ethics Statement

Institutional Animal Care and Use Committee of University of Arkansas approved the study protocol for welfare guidelines and husbandry practices performed in the experimental period. Sampling bird selection: myopathy and non-myopathy birds. The committee is in strict compliance with Public Health Service Policy on Humane Care and Use of Laboratory Animals (PHS Policy), the USDA Animal Welfare Act and Regulations (AWAR), the institutional Animal Welfare Assurance, and the University Policy on Animal Care and Use.

## Author Contributions

PM, CO, and CC brought the research idea, designed the study, and discussed and interpreted the findings. PM conducted the experiment and wrote the manuscript. JW, KH, NS, and AB contributed in performing the experiment and collecting the data. All authors contributed in intellectual contribution.

### Conflict of Interest

The authors declare that the research was conducted in the absence of any commercial or financial relationships that could be construed as a potential conflict of interest.

## References

[1] Emmerson D. Commercial approaches to genetic selection for growth and feed conversion in domestic poultry. Poult Sci. (1997) 76:1121–5. 10.1093/ps/76.8.1121 9251138

[2] Tallentire CW Leinonen I Kyriazakis I Breeding for efficiency in the broiler chicken: a review. Agron Sustain Dev. (2016) 36:66 10.1007/s13593-016-0398-2

[3] Bailey RA Watson KA Bilgili S Avendano S. The genetic basis of pectoralis major myopathies in modern broiler chicken lines. Poult Sci. (2015) 94:2870–9. 10.3382/ps/pev304 26476091 PMC4988535

[4] Kuttappan V Hargis B Owens C. White striping and woody breast myopathies in the modern poultry industry: a review. Poult Sci. (2016) 95:2724–33. 10.3382/ps/pew216 27450434

[5] Wold JP Veiseth-Kent E Høst V Løvland A. Rapid on-line detection and grading of wooden breast myopathy in chicken fillets by near-infrared spectroscopy. PloS ONE. (2017) 12:e0173384. 10.1371/journal.pone.0173384 28278170 PMC5344484

[6] Sihvo H Immonen K Puolanne E. Myodegeneration with fibrosis and regeneration in the pectoralis major muscle of broilers. Vet Pathol. (2014) 51:619–23. 10.1177/0300985813497488 23892375

[7] Kuttappan VA Bottje W Ramnathan R Hartson SD Coon CN Kong B. Proteomic analysis reveals changes in carbohydrate and protein metabolism associated with broiler breast myopathy. Poult Sci. (2017) 96:2992–9. 10.3382/ps/pex069 28499042

[8] Velleman SG Clark DL. Histopathologic and myogenic gene expression changes associated with wooden breast in broiler breast muscles. Avian Dis. (2015) 59:410–8. 10.1637/11097-042015-Reg.1 26478160

[9] Tijare VV Yang F Kuttappan V Alvarado C Coon C Owens C. Meat quality of broiler breast fillets with white striping and woody breast muscle myopathies. Poult Sci. (2016) 95:2167–73. 10.3382/ps/pew129 27081195

[10] Caldas JV Hilton K Boonsinchai N England JA Mauromoustakos A Coon CN. Dynamics of nutrient utilization, heat production, and body composition in broiler breeder hens during egg production. Poult Sci. (2018) 97:2845–53. 10.3382/ps/pey133 29688556

[11] Brouwer E Report of the subcommittee on constants and factors. In Proceedings Energy Metabolism, 3rd Symposium. Troon; London: EAAP Publication 11. Academic press (1965). p. 41–443.

[12] Caldas Cueva JV Calorimetry and Body Composition Research in Broilers and Broiler Breeders (2015).

[13] Kozich JJ Westcott SL Baxter NT Highlander SK Schloss PD. Development of a dual-index sequencing strategy and curation pipeline for analyzing amplicon sequence data on the MiSeq Illumina sequencing platform. Appl Environ Microbiol. (2013) 79:5112–20. 10.1128/AEM.01043-13 23793624 PMC3753973

[14] Schloss PD Westcott SL Ryabin T Hall JR Hartmann M Hollister EB. Introducing mothur: open-source, platform-independent, community-supported software for describing and comparing microbial communities. Appl Environ Microbiol. (2009) 75:7537–41. 10.1128/AEM.01541-09 19801464 PMC2786419

[15] MacLean B Tomazela DM Shulman N Chambers M Finney GL Frewen B. Skyline: an open source document editor for creating and analyzing targeted proteomics experiments. Bioinformatics. (2010) 26:966–8. 10.1093/bioinformatics/btq054 20147306 PMC2844992

[16] Leeson S Predictions for commercial poultry nutrition. J Appl Poult Res. (2008) 17:315–22. 10.3382/japr.2007-00101

[17] Barzegar S Wu S Choct M Swick RA. Factors affecting energy metabolism and evaluating net energy of poultry feed. Poult Sci. (2019). 10.3382/ps/pez554. [Epub ahead of print]. 32416835 PMC7587646

[18] Wolfe RR. Regulation of muscle protein by amino acids. J Nutr. (2002) 132:3219S−24S. 10.1093/jn/131.10.3219S 12368421

[19] Maharjan P Beitia A Weil J Hilton K Owens C Coon C Mixed muscle protein synthesis in Pectoralis major in two broiler strains in relation to woody breast myopathy. Poult. Sci. (2019) 98(E-Suppl. 1):151–52. 10.3920/978-90-8686-891-9_112 PMC7598337 32988542

[20] Patel H Bhardwaj A Physiology, Respiratory Quotient. Treasure Island, FL: StatPearls Publishing LLC (2019). 30285389

[21] Matarese LE. Indirect calorimetry: technical aspects. J Am Diet Assoc. (1997) 97:S154–60. 10.1016/S0002-8223(97)00754-2 9336580

[22] Walsberg G Wolf B. Variation in the respiratory quotient of birds and implications for indirect calorimetry using measurements of carbon dioxide production. J Exp Biol. (1995) 198:213–9. 9317662 10.1242/jeb.198.1.213

[23] Holzapfel WH Wood BJ Lactic Acid Bacteria: Biodiversity and Taxonomy. Hoboken, NJ: John Wiley & Sons (2014).

[24] Gratta F Fasolato L Birolo M Zomeño C Novelli E Petracci M. Effect of breast myopathies on quality and microbial shelf life of broiler meat. Poult Sci. (2019) 98:2641–51. 10.3382/ps/pez001 30668837

[25] Altaher Y Jahromi M Ebrahim R Zulkifli I Liang J *Lactobacillus pentosus* ITA23 and L. acidipiscis ITA44 enhance feed conversion efficiency and beneficial gut microbiota in broiler chickens. Braz J Poult Sci. (2015) 17:159–64. 10.1590/1516-635x1702159-164

[26] Wierzbicki AS. Homocysteine and cardiovascular disease: a review of the evidence. Diabetes Vasc Dis Res. (2007) 4:143–9. 10.3132/dvdr.2007.033 17654449

[27] Ubbink D. Homocysteine—an atherogenic and a thrombogenic factor? Nutr Rev. (1995) 53:323–5. 10.1111/j.1753-4887.1995.tb01486.x 8643214

[28] Romano KA Vivas EI Amador-Noguez D Rey FE. Intestinal microbiota composition modulates choline bioavailability from diet and accumulation of the proatherogenic metabolite trimethylamine-N-oxide. MBio. (2015) 6:e02481–14. 10.1128/mBio.02481-14 25784704 PMC4453578

[29] Andersen G Marcinek P Sulzinger N Schieberle P Krautwurst D. Food sources and biomolecular targets of tyramine. Nutr Rev. (2018) 77:107–15. 10.1093/nutrit/nuy036 30165672

[30] Lincoln TM. Cyclic GMP and mechanisms of vasodilation. Pharmacol Ther. (1989) 41:479–502. 10.1016/0163-7258(89)90127-7 2541452

[31] Arsenian MA. Carnitine and its derivatives in cardiovascular disease. Prog Cardiovasc Dis. (1997) 40:265–86. 10.1016/S0033-0620(97)80037-0 9406679

[32] Papah MB Brannick EM Schmidt CJ Abasht B. Gene expression profiling of the early pathogenesis of wooden breast disease in commercial broiler chickens using RNA-sequencing. PloS ONE. (2018) 13:e0207346. 10.1371/journal.pone.0207346 30517117 PMC6281187

[33] Malila Y Thanatsang K Arayamethakorn S Uengwetwanit T Srimarut Y Petracci M. Absolute expressions of hypoxia-inducible factor-1 alpha (HIF1A) transcript and the associated genes in chicken skeletal muscle with white striping and wooden breast myopathies. PLoS ONE. (2019) 14:e0220904. 10.1371/journal.pone.0220904 31393948 PMC6687142

[34] Sihvo H Airas N Lindén J Puolanne E. Pectoral vessel density and early ultrastructural changes in broiler chicken wooden breast myopathy. J Comp Pathol. (2018) 161:1–10. 10.1016/j.jcpa.2018.04.002 30173852

[35] Mudalal S Babini E Cavani C Petracci M. Quantity and functionality of protein fractions in chicken breast fillets affected by white striping. Poult Sci. (2014) 93:2108–16. 10.3382/ps.2014-03911 24902697

[36] Kuttappan V Shivaprasad H Shaw D Valentine B Hargis B Clark F. Pathological changes associated with white striping in broiler breast muscles. Poult Sci. (2013) 92:331–8. 10.3382/ps.2012-02646 23300297

[37] Petracci M Mudalal S Soglia F Cavani C Meat quality in fast-growing broiler chickens. Worlds Poult Sci J. (2015) 71:363–74. 10.1017/S0043933915000367

[38] Papah MB Brannick EM Schmidt CJ Abasht B. Evidence and role of phlebitis and lipid infiltration in the onset and pathogenesis of Wooden Breast Disease in modern broiler chickens. Avian Pathol. (2017) 46:623–43. 10.1080/03079457.2017.1339346 28609139

